# The Academy’s Pivotal Role in Supporting Public-Private Partnerships to Prevent Chronic Diseases

**Published:** 2009-03-15

**Authors:** Michael A. McRobbie, Lloyd J. Kolbe

**Affiliations:** Indiana University; Indiana University, Bloomington, Indiana

## Introduction

Chronic diseases — such as heart disease, cancer, and diabetes — are the leading causes of death in the United States; they account for 70% of all deaths annually. Chronic diseases also limit daily activities for nearly 10% of Americans. Although these diseases are among the most common and expensive health problems, they are also among the most preventable (1). This growing health care crisis and its attendant economic costs can be addressed only by sharing expertise and resources across sectors.

Transdisciplinary research, education, and service will be vital to preparing professionals in the 21st century to perform their respective and collective roles in improving the well-being of humankind locally, nationally, and globally. Universities have a crucial role to play in assuring the health and economic security of the society that supports them. The academy can become the synapse that connects the research and teaching assets of higher education to the real-life public health needs of communities.

The 3 million employees of our nation's 2,600 four-year, degree-granting institutions serve nearly 18 million students. Universities are well positioned through their interrelated academic functions of research, education, and service to become pivotal partners in public-private efforts to control chronic diseases across sectors (public, not-for-profit, and private), disciplines (public health, social and political sciences, and economics), and levels (local, state, national, and global) (2). The purpose of such efforts, from an economist's perspective, is to maximize population health (3). By way of illustration, we cite examples of efforts undertaken by Indiana University that are conceived to help control chronic diseases.

## Research

Given the fundamental determinants of population health — physical and social environment; individual genetics, biology, behavior, function; disease; and health care (4) — research to improve public health needs to be centered on population health, transdisciplinary, grounded in the fundamental social and economic determinants of health, participatory and inclusive, and intersectoral in design (5).

Scholars have categorized public-private partnerships according to degree of combined governance (equal negotiation capacity vs delegation), nature of activity (consultative vs operational), and goals of the partnership (eg, product-focused vs service-oriented). The limited number of such partnerships that exist worldwide suggests that a minimum set of elements is required to succeed. These elements include commonality of goals, strategies, and values; formation of interpersonal relationships between partnering institutions; creation of value for both partners; concrete guidelines to facilitate feedback and communication; and agreement about criteria to evaluate the extent to which partnerships have achieved goals.

In 2002, Indiana University embarked on such a partnership by developing the Indiana University Life Sciences Strategic Plan (6). The plan, which is designed to foster basic and translational research, observes the following:

While Indiana's educational systems strive to create a well-educated workforce for the 21st century, a more robust and vibrant economy will not be realized if it depends for its construction on a workforce facing chronic health problems. Companies that consider locating in Indiana will take into account the cost of medical insurance and the health of the workforce…. Indiana University should engage in research and education … [and] should continue and expand efforts to inform residents of the state of Indiana about the importance of exercise and proper nutrition. The university should continue and expand efforts within the state of Indiana to reduce the prevalence of smoking, excessive use of alcohol and controlled substances, and risky sexual activities.

Indiana University's research centers, such as the Indiana Prevention Resource Center (http://www.drugs.indiana.edu/), which provides a statewide clearinghouse for prevention information about alcohol, tobacco, and other drugs, and the Rural Center for AIDS/STD Prevention (http://www.iub.edu/~aids/), which develops and evaluates educational materials and programming, have key roles to play in this effort.

## Education

Faculty members across Indiana University's 8 campuses teach courses about public health. Indiana University at Bloomington offers a bachelor of science degree in public health. Indiana University at Bloomington and Indiana University/Purdue University at Indianapolis each offer a master in public health (MPH) degree. Students are often placed in private-sector organizations to complete their internship requirements. Indiana University MPH graduates are increasingly employed by private-sector organizations.

Universities can do more than train only those who will work in public health to create the conditions in which people can be healthy. Professionals in many disciplines (eg, business, social sciences, economics) make critical personal and professional decisions throughout their lives that affect their own health, the health of their families, and the health of the communities in which they live.

Indiana University awards a doctoral minor in public health and is submitting public health foundation courses and intensive public health study-abroad courses for consideration as undergraduate elective courses by its general education course selection committee. These courses may appeal to all undergraduate majors, including business majors; businesses should appreciate the advantages of employees who understand that public health improvements equate with productivity improvements.

At Indiana University we have also begun to analyze the feasibility of establishing a school of public health. As an initial step, a task force on public health is assessing the state's need for a school of public health (7). Such a school would pursue research, education, and service and would work closely with state and local agencies to improve health and economic vitality in Indiana and beyond. Discussions and deliberations following from the report are continuing, as is the evaluation of models at other universities.

## Service

With 21 million students and employees, 4-year, degree-granting universities are uniquely influential worksites from which efforts to prevent chronic diseases can be mounted. Campus environments that encourage students to establish healthy lifestyles result in economic benefits for the university, future employers, and the health care system. Universities can also improve the health of their sizeable workforces and thereby help control health insurance costs. Finally, universities are integral to the communities that surround them and can do much to reduce chronic diseases in those communities, especially in partnership with community businesses. Indeed, the World Health Organization has suggested ways for universities to improve public health (8).

To help prevent chronic diseases among its approximately 100,000 students, Indiana University has implemented diverse interventions. These include working with private-sector vendors to offer more nutritious meals and snacks, implementing health-promoting dormitories, establishing tobacco-free campuses, and providing insurance incentives to help employees avoid tobacco use.

Indiana University is also engaged in AIDS/human immunodeficiency virus (HIV) prevention, testing, and treatment measures on the global stage. Our longstanding partnership with Moi University in Kenya has yielded the Academic Model for Providing Access to Healthcare (AMPATH) (www.medicine.iupui.edu/kenya/hiv.aids.html), which was nominated for a Nobel Peace Prize. One of the largest and most comprehensive HIV/AIDS treatment and prevention programs in the world, AMPATH treats more than 60,000 patients and feeds more than 30,000 people a week.

AMPATH has implemented a robust program to prevent mother-to-child transmission of HIV, including an opt-out testing policy and programs that promote triple antiretroviral therapy for pregnant women and formula feeding of newborns. In summer 2008, the Global Business Coalition announced that it would work with the United States Agency for International Development and AMPATH to roll out the most powerful HIV education, prevention, and case-finding program ever devised: home-based counseling and testing that integrates various means of primary, secondary, and tertiary prevention. In each home, a visiting counselor will conduct a rapid test for HIV, screen for tuberculosis, provide 2 medicated bed nets, and administer deworming medicine to all children.

## Conclusion

Public-private partnerships are most effective when they are structured to achieve a balanced division of labor and resources. Some public health scholars have used the term "collaboration continuum" to characterize the development of such partnerships. These partnerships may begin as a grant at one end; progress to a "transactional stage," in which partners combine resources toward a common goal; and culminate in an "integrative stage," characterized by merging resources to generate a new identity.

Indiana University, like many other universities, has a strong history of successful collaboration with private-sector agencies to develop marketable technologies and services in other disciplines, such as medicine and informatics. We have advanced similar public-private partnerships to help control chronic diseases, as illustrated in the examples above, but we expect to do more. Universities can be powerful partners as they apply their integrated research, education, and service capacities to join sectors and disciplines across geopolitical levels. We can generate the knowledge required to foster progressively more successful public-private partnerships, with not only universities but also many other public and not-for-profit agencies as well. Such knowledge will be the basis for ever more successful efforts to help control chronic diseases.
